# Niche divergence accelerates evolution in Asian endemic *Procapra* gazelles

**DOI:** 10.1038/srep10069

**Published:** 2015-05-07

**Authors:** Junhua Hu, Zhigang Jiang, Jing Chen, Huijie Qiao

**Affiliations:** 1 Chengdu Institute of Biology, Chinese Academy of Sciences, Chengdu 610041, China; 2 Key Laboratory of Animal Ecology and Conservation Biology, Institute of Zoology, Chinese Academy of Sciences, Beijing 100101, China

## Abstract

Ecological niche divergence and adaptation to new environments are thought to play important roles in driving speciation. Whether recently evolved species show evidence for niche divergence or conservation is vital towards understanding the role of ecology in the process of speciation. The genus *Procapra* is an ancient, monophyletic lineage endemic to Asia that contains three extant species (*P. gutturosa*, *P. przewalskii* and *P. picticaudata*). These species mainly inhabit the Qinghai-Tibetan and Mongolian Plateaus, and today have primarily allopatric distributions. We applied a series of geographic information system–based analyses to test for environmental variation and niche divergence among these three species. We found substantial evidence for niche divergence in species’ bioclimatic preferences, which supports the hypothesis that niche divergence accelerates diversification in *Procapra*. Our results provide important insight into the evolutionary history of ungulates in Asia and help to elucidate how environmental changes accelerate lineage diversification.

The role that ecology plays in speciation has received increased attention in recent years^1^,^2^,^3^. A key question in this debate is whether recently evolved organisms show evidence for niche conservation or divergence. Niche conservatism is the tendency of organisms to retain ancestral ecological niche characteristics over time^1^,^2^,^4^,^5^. Niche conservatism was proposed explicitly and tested quantitatively only a decade ago^2^, with the conclusion that ecological niches show considerable conservatism over evolutionary time periods^3^. The degree to which ecological niches are conserved carries implications for a range of ecological and evolutionary phenomena, from the role of ecology in speciation to expected responses of species to climate change^1^,^4^,^6^,^7^,^8^.

At first glance, evidence for niche conservatism appears to be mixed. Considerable structure, however, is evident when the patterns are time-structured. That is to say, recent and short-term events (e.g. species invasions, distributional shifts over relatively short time periods) show a tendency towards conservatism, whereas longer-term events (e.g. differentiation across phylogenies) show a tendency towards breakdown in conservatism^3^. Although niches seem to be generally conserved over time spans relevant to speciation and distributional patterns, niche divergence is also thought to promote diversification in organisms along ecological gradients^6^,^9^. Evidence for niche divergence would support a role for ecological speciation in which divergent natural selection promotes diversification through adaptation to new environments^10^.

The gazelles and allies of the tribe Antilopini belong to the subfamily Antilopinae (Bovidae, Artiodactyla), which are considered one of the most diverse but also least understood and phylogenetically-controversial bovid groups^11^. Within the tribe, the genus *Procapra* is an ancient, monophyletic lineage endemic to Asia. Member of this group share unique characteristics, such as the females are hornless and have only rudimentary facial glands^11^,^12^,^13^. *Procapra* split from the Antilopini 11–12 Mya ago^11^,^14^. The group includes three living species: the Mongolian gazelle (MG, *P. gutturosa*), the Przewalski’s gazelle (PG, *P. przewalskii*), and the Tibetan gazelle (TG, *P. picticaudata*). TG split from the common ancestor of MG-PG approximately 3.46 Mya, while PG-MG split at about 0.88 Mya^15^. These gazelles occupy areas spanning from the Qinghai-Tibetan Plateau (QTP) to the Mongolian Plateau, with the Qilian Mountains as a barrier between populations of PG and TG in the south and MG in the north (Fig. 1)^12^,^16^. PG and TG are flagship ungulates on the QTP, which is characterized by high diversity and endemism of wild ungulates^12^,^17^,^18^. Habitat preferences among *Procapra* gazelles are distinct^13^,^16^. Generally, TG is considered endemic to the QTP, and lives solely in high-elevation areas (~3000–5750 m) with low temperatures, intense solar radiation, and limited oxygen availability^16^. PG occupies lower-elevation areas (but still above 3000 m), frequenting open valleys, grassland steppe, stable sand dunes, and desert-shrub ecotones^12^. MG inhabits the zonal (but not montane, given that individuals avoid hilly areas) arid steppe and plains, occurring at 800–1000 m in areas with low average annual precipitation. Individuals migrate across the vast expanse of Mongolia’s Eastern Steppe as they forage throughout the year^16^,^17^,^19^. All three species have experienced significant population and range declines from poaching, excessive livestock grazing, and habitat loss or fragmentation, such that their distributions are primarily allopatric with only extremely-limited sympatry (e.g. between PG and TG in the Upper Buha River, Qinghai, China^20^,^21^).

Here, we examine whether niche divergence accelerates evolution within the *Procapra*. We test for niche divergence using two stringent tests. The first measures overlap from ecological niche models (ENMs)^22^,^23^ to assess ecological distinctiveness between species. The second is a new analytical approach proposed by McCormack *et al.*^24^ that tests whether species show evidence for niche divergence along multiple niche axes^25^. We consider niche comparisons within a phylogenetic framework^15^,^26^, which provides a broad and multifaceted view of niche variation and differentiation in this clade. Our findings elucidate the potential speciation mechanism in *Procapra* and provide insight into the evolutionary history of ungulates in Central Asia and the QTP^18^. Moreover, our results provide information on how Asian gazelles responded to environmental changes over the past 12 million years across multiple niche dimensions^12^,^15^,^27^.

## Results

### Niche variation and quantification of individual environmental variables

Substantial variation in environmental preference was detected among the three gazelles, with multivariate tests showing significant species effects (Kruskal-Wallis test: *P* < 0.01). However, species did not differ significantly from each other with respect to any of the six *individual* environmental variables (Fig. 2a). MG was associated with the lowest values of T_*min*_ (min mean temperature of the coldest month) and Prec_*anu*_ (annual precipitation), and the highest values of T_*anu*_ (annual mean temperature) and T_*max*_ (max mean temperature of the warmest month). PG was associated with the lowest precipitation values in the driest month (Prec_*dry*_). A discriminant function analysis (DFA) provided further support that the three gazelle pairs occupy markedly different environments (Wilks’s λ: MG-PG, λ = 0.214, *P < *0.0001; MG-TG, λ = 0.178, *P < *0.0001; PG-TG, λ = 0.893, *P < *0.01; Supplementary Table S1). MG and PG differed most in T_*anu*_, whereas MG and TG differed most in T_*max*_. PG and TG differed primarily in T_*min*_.

ENMs predicted the gazelles occur in arid and cold conditions, with low T_*anu*_ (7.7 ± 5.4 SD °C) and relatively little Prec_*anu*_ (536.3 ± 374.8 mm). Prec_*dry*_ was extremely low (3.4 ± 2.6 mm) with a range of 1.1–4.1 mm. Aside from this general similarity, however, species varied strikingly in a number of the bioclimatic variables (Table 1; Fig. 2b). MG fell at the cool end of the spectrum with a T_*anu*_ of 4.0 °C (*vs.* 4.5 °C for PG and 10.4 °C for TG). PG and TG tended to experience low T_*max*_, while MG experienced low T_*min*_. Turning to the precipitation dimensions, overlap for Prec_*dry*_ was greatest for MG-TG (Fig. 2c). Moreover, overlap for half of the bioclimatic variables (T_*anu*_, Prec_*anu*_ & Prec_*wet*_ (precipitation of the wettest month)) was greatest for MG-PG, while it was greatest in T_*max*_ and T_*min*_ for PG-TG. Based on a Principal Component Analysis (PCA) performed on the ENM predictions, projected overlap was broad in PC1 for all species (Fig. 3). TG possessed the broadest bioclimatic niche space, which overlapped entirely with the other two gazelles in PC1 and PC2. Significant differences in the bioclimatic envelopes of the three species were found using a Multivariate Analysis of Variance (MANOVA; Wilk’s λ = 0.43, F_4, 143934_ = 18812.2, P < 0.01). Along the two first PCs, species exhibited statistically significant bioclimatic separation (X axis: F_2, 71968_ = 3081.3, P < 0.01; Y axis: F_2, 71968_ = 34671.9, P < 0.01), with PC1 explaining 59.96% and PC2 only explaining 26.26% of the variance, respectively.

### Testing niche divergence and conservatism

We tested for niche divergence and conservatism on independent niche axes using a multivariate analysis of the raw bioclimatic data. Four PCs were identified that explained 99.56% of the total variance and availed themselves to biological interpretation (Table 2). Niche axes associated with annual precipitation and temperature variables explained most of the variance in PC1 and PC2, but were also highly correlated with geographical variables (longitude and latitude). Evidence for niche divergence was detected in most tests (eight of 12). Specifically, the MG-PG and MG-TG species pairs were characterized primarily by divergence. The PG-TG pair showed little evidence for niche divergence, which was suggested for only one of the four niche axes (Table 2).

ENM-based background tests for reciprocal comparisons of each species-pair showed support for niche divergence when compared to null models of background divergence (Fig. 4). Eight of 12 comparisons deviated from the null background expectation. One reciprocal comparison (MG and PG) revealed significant evidence for niche divergence with respect to null distributions regardless of the measure of similarity used (i.e. *D* or *I*). Niche divergence was detected in the comparisons of TG versus MG’s background, and TG versus PG’s background, but the opposite comparisons did not deviate from null expectation.

## Discussion

We tested for niche divergence within *Procapra* gazelles endemic to Asia. Understanding the degree to which closely related and/or partly sympatric species diverge in their niche traits is important for understanding the mechanisms underpinning broad-scale biogeographic patterns^1^,^2^,^25^,^28^, and may elucidate the role that ecology plays in speciation. That is to say, niche differentiation may accelerate evolution as predicted under ecological speciation.

We found evidence for niche differentiation among *Procapra* species using both a Kruskal-Wallis test and DFA. Moreover, our results indicate strong interspecific variation in environmental requirements^29^. We rejected the hypothesis that *Procapra* species-pairs are distributed in identical environmental space via niche-identity tests^29^. In addition, more than half of the background similarity tests indicate greater divergence among species than would be expected from their available habitat. This suggests there is substantial niche divergence among *Procapra* gazelles (Table 2; Fig. 4).

Ecology has been posited to play an important role in lineage diversification and speciation when niches show little overlap between closely related species^30^,^31^. However, geographical isolation alone will typically not drive ecological divergence. Strong environmental (e.g. bioclimatic and physiological) differences often need to be associated with the distinct geographical ranges for divergence to occur^32^. This seems to be the case for species-pair MG-PG, which is distributed allopatrically and exhibits strong niche divergence. Geographic isolation and differing environmental conditions likely contributed to the observed divergence in niche traits between these species, which in turn promoted reproductive isolation and genetic diversity^15^.

Interestingly, niche divergence was often indicated for only one of the niche axes using the multivariate PCA method proposed by McCormack *et al.*^24^, a pattern which contrasts with the results from the ENM-based analyses for species-pair PG-TG. The discrepancy, however, is not surprising, given that ENM-based approaches have the potential to overlook smaller, but nonetheless important, ecological differences^24^. That is to say, ENMs estimate the niche by considering varying contributions from many environmental factors jointly, which is akin to testing niche differences along a single PC axis with different variable loadings. The multivariate PCA method, such as the one proposed by McCormack *et al.*^24^, provides more detailed information on niche divergence, and is in better keeping with the Hutchinsonian idea of the niche as a multidimensional hypervolume^33^, in which some axes will diverge while others remain conserved. We found that several species-pairs diverged in only one to three of the four PCA axes (see Table 2 for details). These complicated outcomes are reasonable, as it is important to remember that even where environmental niches differ significantly, the change could be caused by other factors such as the presence of competitors^7^,^34^. Therefore, although niche differentiation may have been caused by divergent selection on the environmental variables themselves, there may well be other explanations for realized species ranges and other drivers of divergence. Nevertheless, when the bulk of evidence is considered, a general pattern emerges, in that the niche seems to be conserved across PG-TG and differentiated across MG-TG and MG-PG. Species within *Procapra* occupy dramatically different climates and topography, and thus it is not surprising to find disparate patterns suggesting different modes of species divergence with respect to the ecological niche.

Speciation within *Procapra* was thought to be closely tied to the uplift of the QTP, although fossil material is limited^12^,^15^,^26^. Based on isotope analyses in the Kunlun Basin, climate in the QTP during the Pliocene (2–3 Ma) was suggested to be milder and wetter than at present^35^. These conditions, combined with the uplift of the QTP, may have led to diversification of *Procapra*^12^,^13^,^36^. That being the case, what properties of organisms and their environments lead to the evolution of discrete species^37^? Although this is an abstract and difficult question, some aspects of it can be demonstrated, given that rapid niche evolution is linked with speciation^38^. The maintenance of organisms in geographically-distinct areas must be due, at least in part, to the conservatism of niche preferences through natural selection against individuals that disperse out of their current niche (e.g. Wiens^39^). While niche conservatism may exert a powerful influence on the distribution of organisms, it is still possible for organism to exhibit divergence in environmental preferences on short evolutionary time scales^40^.

When testing for the role of ecology in diversification using large-scale ecological data, there is an important caveat that niche axes important to divergent selection pressures might be overlooked^24^. This is especially relevant because divergence during ecological speciation is often driven by strong differences along a single niche axis^41^. For the niche overlap of a single variable explored here, the greatest values of overlap for different climate variables differed across the species pairs (Fig. 2c). This issue is related to the problem of scale^42^, where niche characteristics that are heterogeneous at local scales are expected to drive ecological speciation because they capture variation in resources, which are often important to divergent selection^24^. The fact that PG and TG have similar social structure, dietary composition and activity budgets, but differ in the utilization of core home ranges and some habitat factors within sympatry^21^ can result in reproductive isolation between individuals. Due to restricted gene flow among populations of these gazelles^15^,^43^, it is likely that, after sympatric speciation between the ancestor of MG-PG and TG and geographic separation between MG and PG, niche divergence accelerated evolution in *Procapra*.

The QTP is characterized by a wide array of complex and heterogeneous habitats supporting the most endemic-rich temperate flora in the world^44^ and provides a model ecosystem for investigating speciation^36^,^45^,^46^. The Late Cenozoic uplift of the QTP provides novel ecological opportunities and seems to be a driving force for shaping recent genetic structure and biodiversity within the region^47^. Although we studied a small radiation within the Antilopinae, the framework used in this study for diversification involved the establishment of closely-related species with largely disjunct geographic ranges^48^, which is ideal for elucidating evolutionary relationships. Moreover, the patterns uncovered here may be useful in exploring patterns of diversity in other vertebrate groups on the steppes of Central Asia and the QTP^18^.

## Methods

### Species distribution patterns and occurrence data

MG is distributed across Inner Mongolia of China and eastern Mongolia and adjacent areas of Russia, with smaller populations in central and western Mongolia^19^,^49^. PG is arguably among the most endangered large mammals on Earth, surviving in remnant populations restricted to small portions of its former range in the vicinity of Qinghai Lake^12^,^50^. Historically, PG occurred in semiarid grassland steppes of the Chinese provinces of eastern Qinghai, Inner Mongolia (Ordos and Alashan plateaus), Gansu (Hexi Corridor), Ningxia (Helan Mountains), and Shanxi^12^,^51^. However, environmental changes have severely altered its current distribution and continue to pose a threat to the species’ survival^27^. Although TG is one of most widespread ungulates on the QTP, its geographic range has also been fragmented in several patches (e.g. Kekexili, Arjin Shan, Chang Tang, Ruoergai, and Mazongshan)^36^,^43^, with small peripheral populations in Ladakh and Sikkim^16^,^17^,^52^.

**Figure 1:**
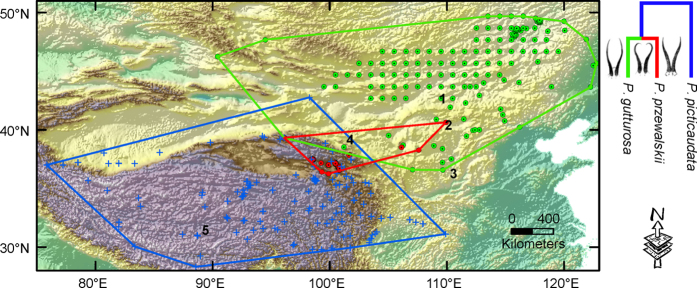
Spatial occurrence records for the three species in *Procapra*: *P. gutturosa* (green circles), *P. picticaudata* (blue circles) and *P. przewalskii* (red crosses). Arabic numerals indicate locations mentioned in this study: 1, Mongolian Plateau; 2, Hetao Ordos middle high plain; 3, Loess Plateau; 4, Qilian Mountains; 5, Qinghai-Tibetan Plateau. The polygons represent the minimum convex polygons that circumscribed the occurrences for each species. This figure is generated based on elevation data from the CGIAR International Research Centers ( http://srtm.csi.cgiar.org/) using ArcGIS 9.2 (ESRI, Redland, CA).

**Figure 2:**
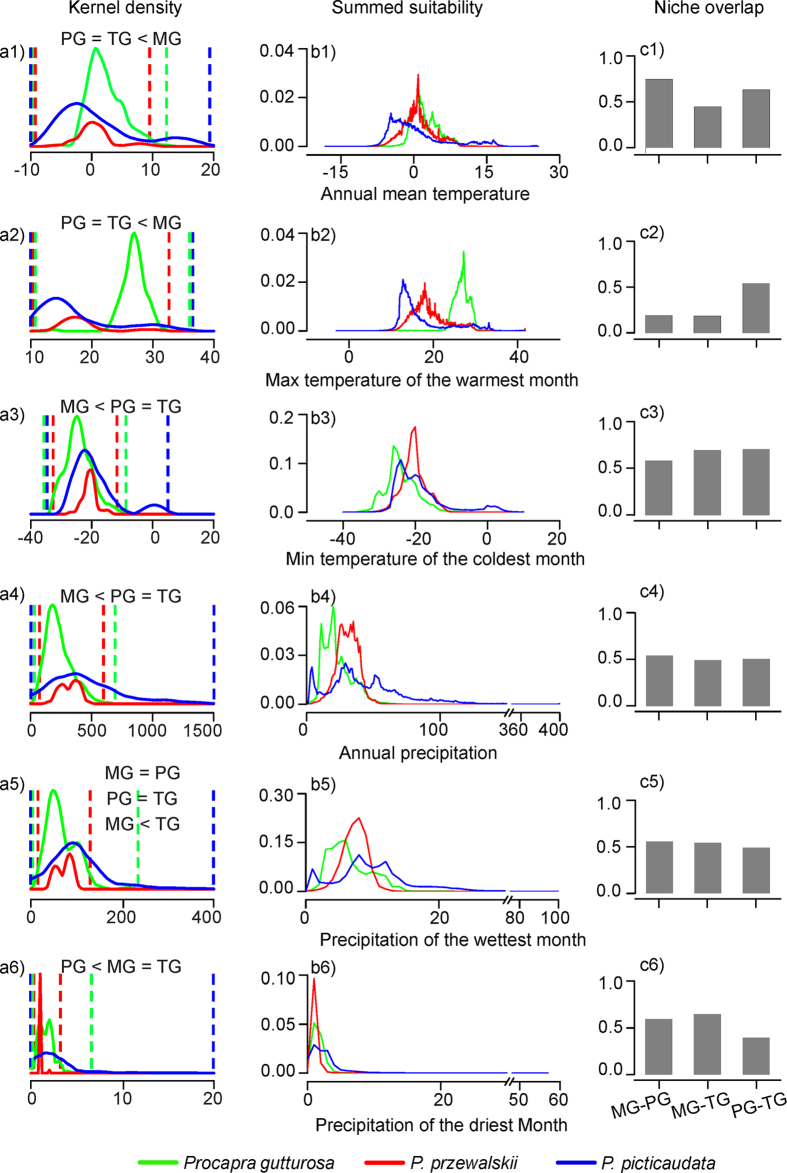
Kernel density plots (**a**), predicted niche occupancy (**b**) and niche overlap (**c**) with respect to each bioclimatic variable for the three species in *Procapra*. In panel (**a**), differentiation among species is evaluated by the Kruskal-Wallis test based on the occurrence records, with results indicated in each plot. The dashed vertical lines show the range of variable in the given areas accessible to each species. In panel (**b**), predicted suitability (Maxent “raw probabilities”) is summed according to the bioclimatic variable with which it is associated. Suitability is rescaled for each variable and species. In panel (**c**), niche overlap in each variable is quantified by comparing predicted niche occupancy profiles following Evans *et al.*^64^. *P. gutturosa*, *P. przewalskii*, and *P. picticaudata* are denoted as MG, PG and TG, respectively. T_*anu*_, annual mean temperature; T_*max*_, max temperature of the warmest month; T_*min*_, min temperature of the coldest month; Prec_*anu*_, annual precipitation, Prec_*anu*_, precipitation of the wettest month; Prec_*dry*_, precipitation of the driest month. Temperature (°C), Precipitation (mm).

**Figure 3:**
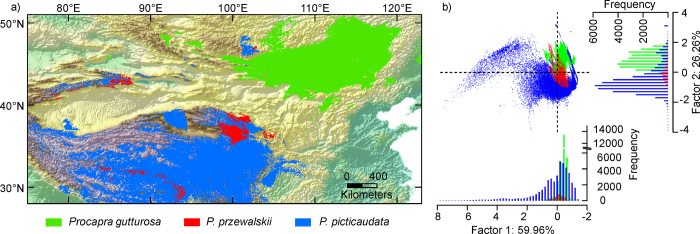
Projected distributions (**a**) and PCA (Principal Component Analysis) plots (**b**) from the ecological niche models for the three species in *Procapra*. PCA plots are based on a logistic climatic suitability value representing the 10^th^ percentile training presence threshold of actual occurrence records of each species. Panel (**a**) is generated based on the projected distributions of each species using ArcGIS 9.2 (ESRI, Redland, CA).

**Figure 4:**
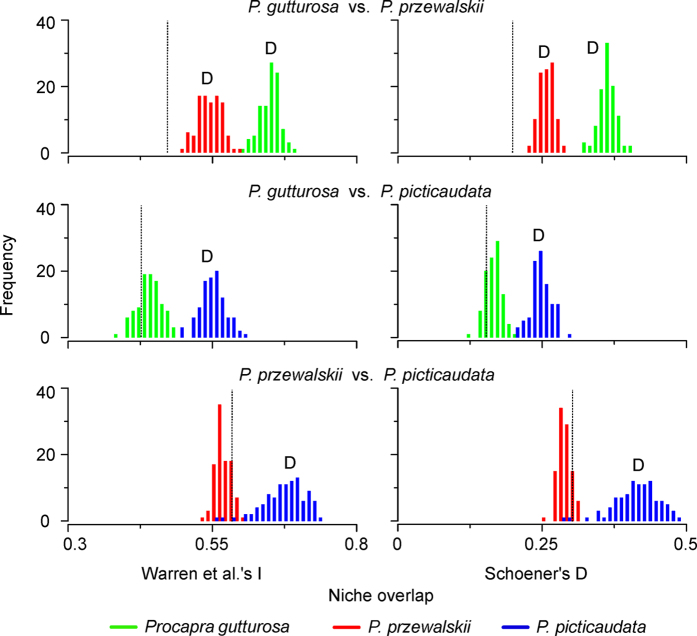
Tests of niche divergence and conservatism from ecological niche models. Niche overlap values (black dotted lines) of I (left panels) and D (right panels) are examined and compared to a null distribution of background divergence. Each pairwise comparison produces two reciprocal analyses, one in which the niche model for species A is compared to a niche model generated from random points from species B’s geographic range and vice versa (hence, the two shaded distributions in each plot). Overlap values smaller than the null distribution support niche divergence (D), whereas larger values indicate niche conservatism (C).

We obtained occurrence data for *Procapra* gazelles from diverse sources in order to characterize the entirety of their distributional ranges (for details see Hu and Jiang^29^). We employed a spatial filter to occurrence data so that only one record remained within each grid cell at a spatial resolution of 8 × 8 km. In total, this resulted in 322 georeferenced occurrences across the three species (156 for MG, 34 for PG, and 132 for TG, respectively; Fig. 1).

### Environmental variables

Environment variables for use in ENMs should be selected on a taxon-specific basis^53^. We used only climatic data, given that predictive power does not improve substantially when variables other than climate are included^54^. Our aim was to explore climatic niche variation and to model the suitable areas for *Procapra* on both large temporal and spatial scales, and as such, we prioritized variables that change slowly through time^55^. Furthermore, we selected only those variables thought to be important to the ecology of *Procapra*^12^,^20^. *Procapra* gazelles seem to be limited by annual and extreme temperatures and precipitations^20^. Indeed, the severity of winter weather, which is often correlated with reduced food availability and quality that would dampen reproductive rates and increase mortality of young, was found to be negatively associated with population size and survival from summer to winter^12^,^20^,^56^. Consequently, we selected six bioclimatic variables that describe surface averages for temperature and precipitation and potentially biologically-limiting extremes from the WorldClim database^57^. These variables included T_*anu*_, T_*max*_, T_*min*_, Prec_*anu*_, Prec_*wet*_, and Prec_*dry*_. Each variable was converted from the original spatial resolution (30”) to 8 × 8 km resolution in ArcGIS 9.2 (ESRI, Redland, CA) to balance the spatial resolution of the occurrence records^58^,^59^.

### Niche variation and quantification on individual environmental variable among species

To assess observed ecological niche differentiation between species, we attached bioclimatic variables values to all occurrences and examined species-level divergence along each variable by means of nonparametric Kruskal-Wallis tests. Kernel density plots were used to visualize species’ distributions across each variable. Next, for each species-pair, relative contributions of the variables were evaluated using a DFA, and Wilks’s λ was used to test the null hypothesis that two species have identical means for the specific variables (See solid lines in Supplementary Fig. S1).

We also assessed niche divergence using models of species’ niche attributes. We extracted suitable environmental conditions from these niche models and repeated the above process for each variable (See dashed lines in Supplementary Fig. S1). Species’ niches were quantified using a maximum entropy algorithm implemented in Maxent 3.3.3 k^60^. Maxent is a presence-background technique that estimates suitability *via* an index of similarity that resembles a heterogeneous point process or logistic regression function^60^,^61^. Maxent performs well with small datasets^62^,^63^ and satisfies a set of constraints representing incomplete information on the distribution and, subject to those constraints, predicts approximate distributions from presence data^60^. Model settings were as follows: 10 bootstrap replicates, evaluation of predictive power with 20% stochastic occurrences, and 10,000 background points. All other parameters were set to default^61^. We focused on the logistic output for ease of interpretation^61^. Suitable area for the species was defined on a Boolean (presence/absence) map that was thresholded from continuous suitability outputs based on the 10th percentile training presence value of the actual occurrences of each species.

To quantify species’ tolerances of climatic niche dimensions, we tabulated Maxent probability distributions with respect to each original bioclimatic variable to produce unit-area histograms of suitability. These histograms illustrate predicted occupancy with respect to each variable for each species^64^. Niche overlap in each variable was quantified by comparing predicted climate occupancy profiles following Evans *et al.*^64^, with the formula 
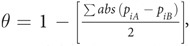
, where, *p*_*i*A_ and *p*_*i*B_ are total predicted suitability at a given value (*i*) of a particular variable for species A and B, respectively.

Finally, we extracted the values of climatic variables within suitable areas for species and conducted a PCA to normalized data for all variables corresponding to each distribution, without *a priori* designation of species. We applied a MANOVA, using the principal components (PCs) as dependent variables and species as categorical variables, to indicate differences in the climatic envelopes among the three species.

### Testing niche divergence and conservatism

Niche divergence between species can result because of actual niche differences or because of spatially-autocorrelated environmental variation^24^. We thus focused on values associated with the occurrences of species compared to those associated with random points from within the region inhabited by or accessible to the species^65^. This process distinguishes the divergences resulting from simple spatial autocorrelation caused by geographic distance from true niche divergence that occurs because two species occupy different habitats^7^,^24^,^65^. Sequentially, to eliminate confounding effects of spatial autocorrelation in bioclimatic variables, we employed both an occurrence-based (i.e. niche-space-based) multivariate test^24^ and an ENM-based background similarity test^22^ to quantify niche divergence versus conservatism among species in *Procapra* (Supplementary Fig. S2).

We drew data from occurrences and from 1000 random points within the accessible range of each species in ArcGIS 9.2 (ESRI, Redlands, CA). Bioclimatic variables were reduced via a PCA of the correlation matrix. We then examined correlations between the PCs and longitude and latitude by a nonparametric correlation test in SPSS 16 (SPSS Inc., Chicago). We retained the first four PCs that explained a modest portion of the total variance (>3%)^24^, and were used as the observed niche values in comparisons with background points. For each PC, comparing observed niche divergence (d_*n*_) to background divergence (d_*b*_), we tested niche divergence and conservatism against a null model of background divergence (d_*b*_ = d_*n*_)^24^. Niche conservatism is supported if d_*b*_ > d_*n*_, whereas niche divergence is supported if d_*b*_ < d_*n*_ and if the observed niche divergence itself (d_*n*_) is significant (based on a t-test). This test provides more detailed information about niche divergence by identifying axes along which the species have diverged, and is useful for detecting environmental variables strongly associated with niche divergence. The method is similar to other approaches that compare divergence in niche space to divergence among targeted absence locations^7^ or visualizes niches within available environmental spaces^66^. Unlike other approaches, however, it explicitly addresses spatial autocorrelation in environmental data, using a null model to establish a baseline expectation for the amount of divergence between allopatric regions^24^. In the reduced PCs, d_*n*_ and d_*b*_ were computed as the differences between the mean scores of 75% random samples from the occurrence records of the two compared species (d_*n*_) and from the background points of the two compared backgrounds (d_*b*_), respectively. We generated distributions of d_*n*_ and d_*b*_ with 1000 random samples, and compared the mean of d_*n*_ to the 95% confidence interval of d_*b*_ to determine significance. These analyses were performed in Systat 13 (SYSTAT Software, Inc. 2009).

The ENM-based background similarity test examines whether observed niche divergence is larger or smaller than differences expected based on the differences in environmental characteristics of the two respective accessible areas^22^. To test the null hypothesis that niches are divergent only to the degree that background environments differs, we calculated two niche overlap indices (*D* and *I*) among ENMs for each species-pair, and used background randomization procedures in ENMtools (Warren *et al.* 2010)^23^ to build a null distribution for comparison. This method compares observed niche overlap values to a null distribution of 100 overlap values generated by comparing the ENM of a focal species to ENMs based on random samples from across the accessible area of the other species^22^. The method tests whether pairs of species are more or less ecologically divergent than would be expected from the differences of environments between their accessible areas. Each test was performed in reciprocal directions for each pair of species. We drew random points from the background within the minimum convex polygon (MCP) that circumscribed the occurrences for each species using the Hawth’s Tools in ArcGIS 9.2 (ESRI, Redlands, CA; for detalis see Warren *et al.*^22^). The number of background random points used was equivalent to the sample size available for the species from whose accessible area points were drawn.

## Author Contributions

J.H. and H.Q. conceived the study; J.H. and H.Q. conducted the analyses; J.H., Z.J., J.C. and H.Q. wrote the paper.

## Additional Information

**How to cite this article**: Hu, J. *et al.* Niche divergence accelerates evolution in Asian endemic Procapra gazelles. *Sci. Rep.* **5**, 10069; doi: 10.1038/srep10069 (2015).

## Supplementary Material

Supporting Information

## Figures and Tables

**Table 1:** Weighted mean values of the bioclimatic variables for species in *Procapra* as predicted by the niche models (T = temperature in °C; P = precipitation in mm) ±SD.

Species	T_*anu*_	T_*max*_	T_*min*_	Prec_*anu*_	Prec_*wet*_	Prec_*dry*_
*Procapra gutturosa*	4.0 ± 1.6	26.7 ± 1.6	−24.6 ± 3.4	249.1 ± 87.9	73.0 ± 26.9	1.9 ± 0.8
*P. przewalskii*	4.5 ± 2.4	18.8 ± 3.3	−19.9 ± 3.1	327.6 ± 70.7	76.6 ± 15.6	1.1 ± 0.3
*P. picticaudata*	10.4 ± 5.4	17.5 ± 5.5	−18.8 ± 6.8	641.1 ± 387.9	143.7 ± 95.0	4.1 ± 2.8

*T_*anu*_, annual mean temperature; T_*max*_, max mean temperature of the warmest month; T_*min*_, min mean temperature of the coldest month; Prec_*anu*_, annual precipitation; Prec_*wet*_, precipitation of the wettest month; Prec_*dry*_ , precipitation of the driest month.

**Table 2:** Divergence on independent niche axes between species pairs of *Procapra*.

	**Species pair**	**PC1**	**PC2**	**PC3**	**PC4**
*P. gutturosa* - *P. przewalskii*	*d*_n_^a^	**0.352 D**	**0.214 D**	**1.785 D**	**0.191 D**
	*d*_b_ (95% null distribution)	0.288-0.294	0.205-0.211	1.294-1.296	0.059-0.062
*P. gutturosa* - *P. picticaudata*	*d*_n_^a^	**1.056 D**	**0.151 D**	**1.933 D**	**0.732 C**
	*d*_b_ (95% null distribution)	0.415-0.424	0.129-0.138	1.567-1.570	0.831-0.834
*P. przewalskii* - *P. picticaudata*	*d*_n_^a^	**0.704 C**	**0.365 D**	**0.148 C**	**0.542 C**
	*d*_b_ (95% null distribution)	0.706-0.714	0.337-0.346	0.273-0.275	0.771-0.773
Percentage of variance explained	47.79	40.90	6.96	3.91	
Top-loading variables	Annual precipitation	Annual mean temperature	Max mean temperature of the warmest month	Precipitation of the wettest month	
Correlation longitude	-0.09**	0.372**	0.563**	0.576**	
Correlation latitude	0.344**	0.04*	0.827*	0.218*	

Bold values indicate significant niche divergence (D) or conservatism (C) compared to a null distribution based on background divergence between their respective geographic ranges. To be divergent, niche values must also differ significantly between the two species.

^a^Significant observed niche divergence (*d*_n_) between species pair is detected for all principal component (PC) axes shown (*t*-test: *P* < 0.05).

^**^P < 0.01 for correlations between PC axes and geographical variables.
