# Supplementary material for: Niche divergence accelerates evolution in Asian endemic Procapra gazelles
Source: Sci Rep. 2015 May 7;5:10069. doi: 10.1038/srep10069 (PMC4423425; doi:10.1038/srep10069)
Supplement: Supporting Information [file srep10069-s1.doc]

Supplementary information for:

**Niche divergence accelerates evolution in Asian endemic *Procapra* gazelles**

Junhua Hu^1, 2^, Zhigang Jiang^2*^, Jing Chen^2^ & Huijie Qiao^2*^

1 Chengdu Institute of Biology, Chinese Academy of Sciences, Chengdu 610041, China

2 Key Laboratory of Animal Ecology and Conservation Biology, Institute of Zoology, Chinese Academy of Sciences, Beijing 100101, China


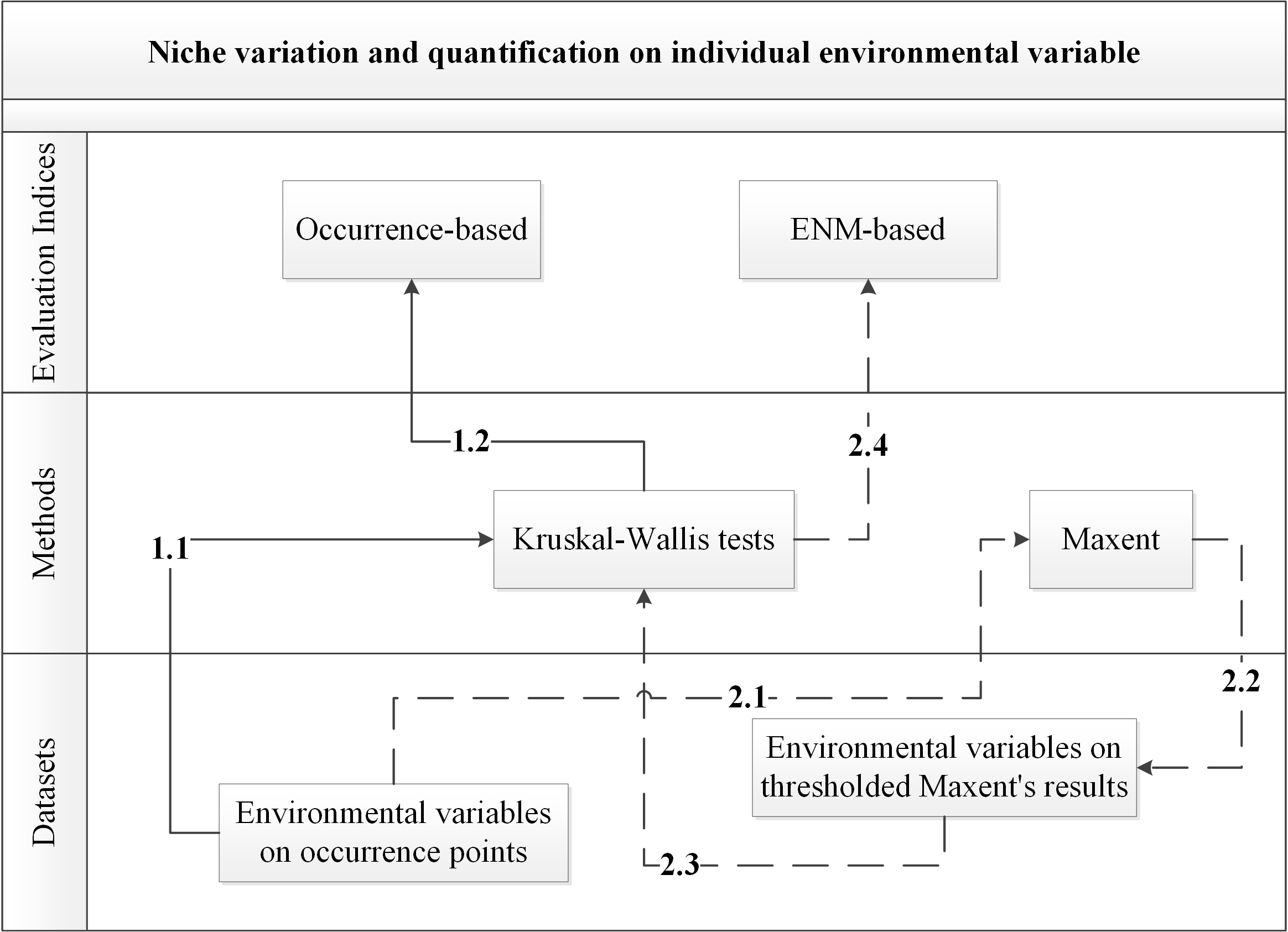


**Supplementary Figure S1** The analysis process to for quantify the niche variation on individual environmental variable. The solid lines with arrows show the occurrence-based analysis process, and ENM-based analysis process is shown with the dash lines. The labels on the lines are the analysis steps.

*
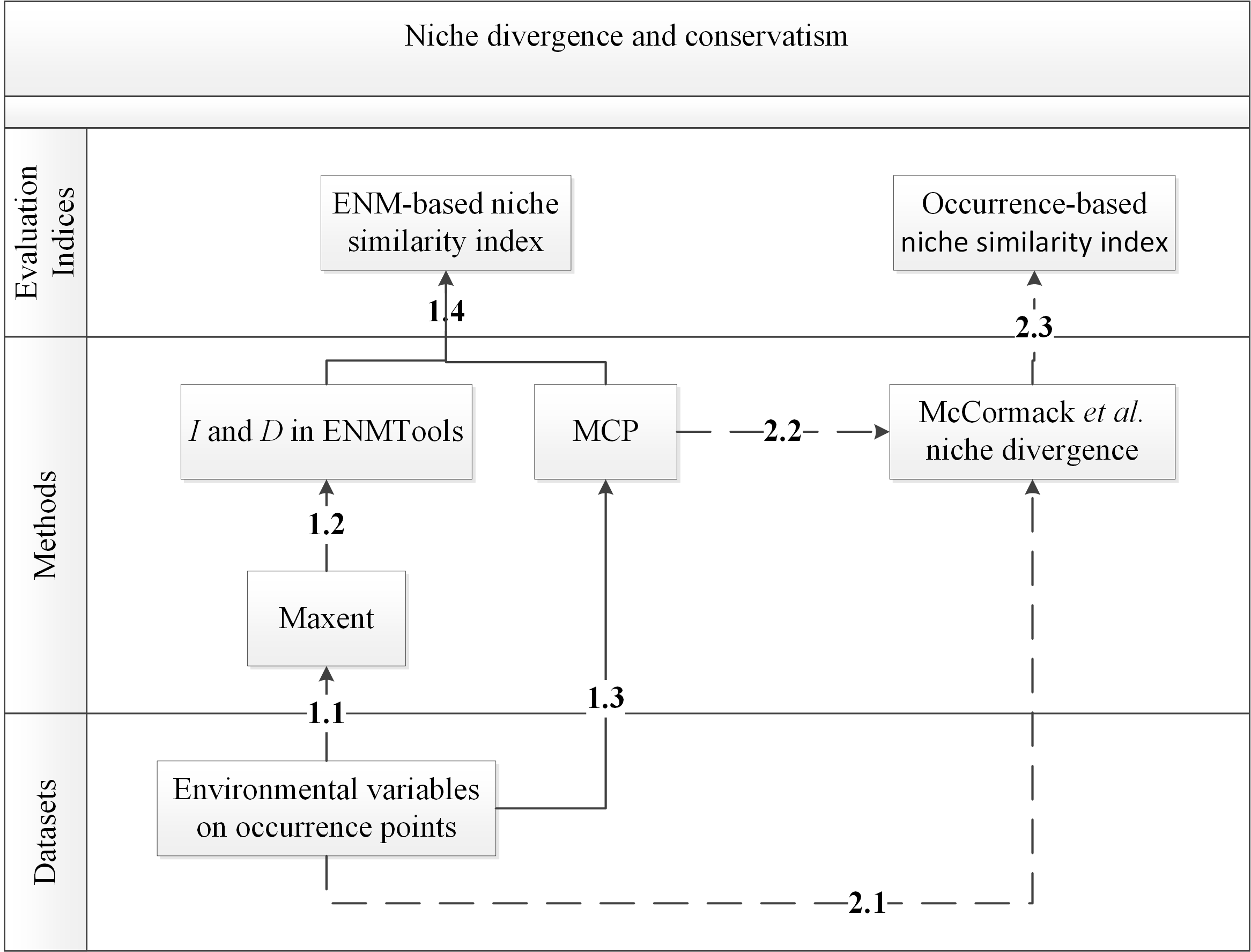
*

**Supplementary Figure S2** The analysis process to for quantify the niche divergence and conservatism on all the environmental variables. The solid lines with arrows show the occurrence-based analysis process, and ENM-based analysis process is shown with the dash lines. The labels on the lines are the analysis steps.

**Supplementary Table S1**  The six bioclimatic variables used in this study and their contributions in discriminant function analysis (DFA) in pairwise comparisons of *Procapra* species

| Environmental variable | *Procapra gutturosa*-*P. przewalskii* | *P. gutturosa*-*P. picticaudata* | *P. przewalskii*-*P. picticaudata* |
| --- | --- | --- | --- |
| Annual mean temperature | **-2.249** | -1.888 | -0.552 |
| Max mean temperature of the warmest month | 1.795 | **2.27** | -0.938 |
| Min mean temperature of the coldest month | 1.297 | -0.129 | **1.344** |
| Annual precipitation | -0.93 | -0.336 | -1.004 |
| Precipitation of the wettest month | 0.894 | 0.708 | 0.23 |
| Precipitation of the driest month | 0.321 | -0.127 | 1.113 |
| DFA (Wilks’s λ) | 0.214 | 0.178 | 0.893 |
| DFA (*P*) | <0.01 | <0.01 | 0.01 |

Note: Values in boldface are for the most significant variables for each comparison.
